# Case report: Early use of whole exome sequencing unveils HNRNPU-related neurodevelopmental disorder and answers additional clinical questions through reanalysis

**DOI:** 10.3389/fgene.2024.1380552

**Published:** 2024-05-23

**Authors:** Erika Nicole Dreikorn, Christine Munro, Natasha Robin Berman, Amina Kunovac, Daniel Bellissimo, Mylynda B. Massart

**Affiliations:** ^1^ Primary Care Precision Medicine Clinic, UPMC, Pittsburgh, PA, United States; ^2^ Department of Family Medicine, University of Pittsburgh, Pittsburgh, PA, United States; ^3^ UPMC Genome Center, Pittsburgh, PA, United States; ^4^ UPMC Clinical Genomics Laboratory, Pittsburgh, PA, United States

**Keywords:** whole exome sequencing (WES), precision medicine, neurodevelopmental disorder (NDD), *HNRNPU* gene mutation, diagnostic odyssey, clinical utility, interdisciplinary collaboration, genome sequencing technologies

## Abstract

This case report chronicles the diagnostic odyssey and resolution of a 27-year-old female with a complex neurodevelopmental disorder (NDD) using Whole Exome Sequencing (WES). The patient presented to a precision medicine clinic with multiple diagnoses including intellectual disability, autism spectrum disorder (ASD), obsessive-compulsive disorder (OCD), tics, seizures, and pediatric autoimmune neuropsychiatric disorders associated with streptococcal infections (PANDAS). Although this patient previously had chromosomal microarray and several single-gene tests, the underlying cause of this patient’s symptoms remained elusive. WES revealed a pathogenic missense mutation in the *HNRNPU* gene, associated with HNRNPU-related neurodevelopmental disorder (HNRNPU-NDD) and developmental and epileptic encephalopathy-54 (DEE54, OMIM: # 617391). Following this diagnoses, other treating clinicians identified additional indications for genetic testing, however, as the WES data was readily available, the clinical team was able to re-analyze the WES data to address their inquiries without requiring additional tests. This emphasizes the pivotal role of WES in expediting diagnoses, reducing costs, and providing ongoing clinical utility throughout a patient’s life. Accessible WES data in primary care settings can enhance patient care by informing future genetic inquiries, enhancing coordination of care, and facilitating precision medicine interventions, thereby mitigating the burden on families and the healthcare system.

## Introduction

The traditional diagnostic pathway for patients with neurodevelopmental disorders (NDD) often involves the lengthy and expensive process of diagnostic testing, which puts immense burden on the patient, their family, and the healthcare system (Vissers et al., 2017). The cumbersome diagnostic pathway can often be navigated more efficiently with the use of whole exome sequencing (WES). WES provides the sequencing resolution necessary to inform current and future medical management for patients.

WES and whole genome sequencing (WGS) are increasingly used to decrease the length of a patient’s diagnostic journey for complex phenotypes; early use of WES can significantly decrease the number of tests and the costs associated with identifying the underlying causes for a patient’s symptoms (Srivastava et al., 2014; Vissers et al., 2017; Clark et al., 2018; Manickam et al., 2021; Runheim et al., 2023). In this case, WES was used for a patient initially presenting with intellectual disability (ID) and autism spectrum disorder (ASD), which identified a pathogenic variant that is likely causal for the patient’s phenotype. Additional genetic clinical indications were evaluated by reanalyzing the WES data, which informed two subsequent indications and avoided the need for additional testing. This case highlights the importance of early WES utilization with future coordination of care being performed in a primary care setting, where the results can be leveraged for informed, coordinated, and lifelong patient care. The coordination aspect is particularly crucial, emphasizing how primary care facilitates integrated care, preventing the repetition of tests that might occur in siloed specialty care settings due to potential gaps in information sharing within the Electronic Health Record (EHR) system.

## Case presentation

**FIGURE 1:**
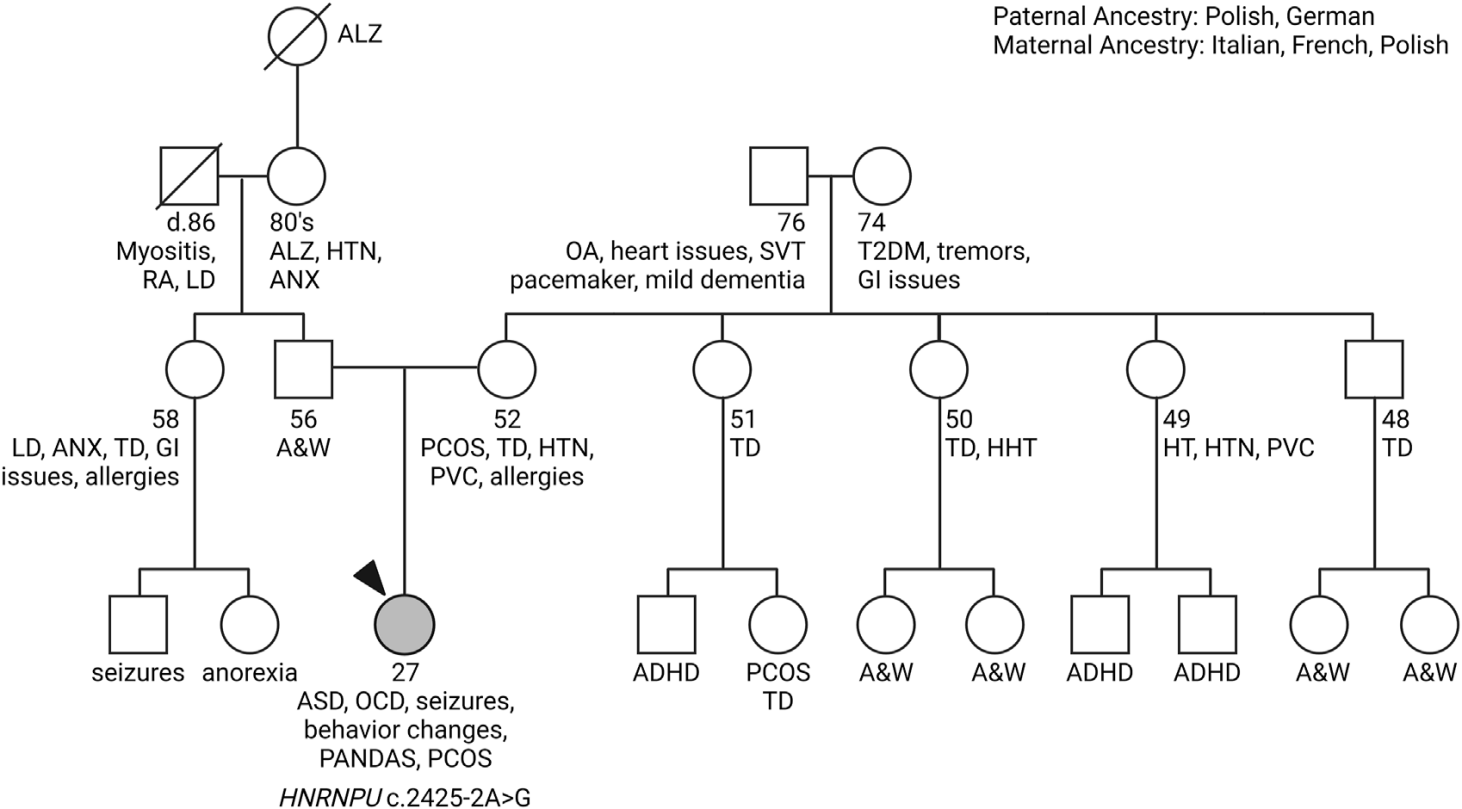
Primary Care Pedigree. At the PCPM clinic, genetic counselors meticulously compile comprehensive family histories, encompassing diverse medical conditions. This thorough examination enables healthcare providers to pinpoint specific areas warranting further genetic investigation. Analyzing the proband’s pedigree, supplied by the patient’s mother, reveals a notable familial prevalence of thyroid disease and heart conditions. However, there is a distinct absence of intellectual disability or neurodevelopmental disorders present in the family, hinting that there is a novel reason for the patient’s symptoms. Arrow indicates proband (patient). Abbreviations: ADHD, attention deficit hyperactivity disorder; ALZ, Alzheimer’s disease; ANX, anxiety; ASD, autism spectrum disorder; A&W, alive and well; HHT, hereditary hemorrhagic telangiectasia; HT, Hashimoto’s thyroiditis; HTN, hypertension; LD, learning difficulty; OA, osteoarthritis; OCD, obsessive-compulsive disorder; PANDAS, pediatric autoimmune neuropsychiatric disorders associated with recurrent streptococcal infections; PCOS, polycystic ovary syndrome; PVC, premature ventricular contraction; RA, rheumatoid arthritis; T2DM, Type II diabetes mellitus; TD, thyroid disease. Created with BioRender.com.

A 27-year-old female was referred by neurology to a precision medicine clinic for consideration of WES to look for an underlying genetic component to her intellectual disability and neurodevelopmental difficulties such as autism spectrum disorder (ASD), obsessive-compulsive disorder (OCD), tics, seizures, and pediatric autoimmune neuropsychiatric disorders associated with streptococcal infections (PANDAS). PANDAS was suspected at age 22 after recurrent Streptococcal infections, when the patient developed encephalopathy. Other clinical presentations included photophobia, a small pituitary gland, polycystic ovarian syndrome (PCOS), amenorrhea, early menopause, and anosmia. The patient also had intermittent elevated iron labs associated with iron overload. See full pedigree (Figure 1).

**FIGURE 2:**
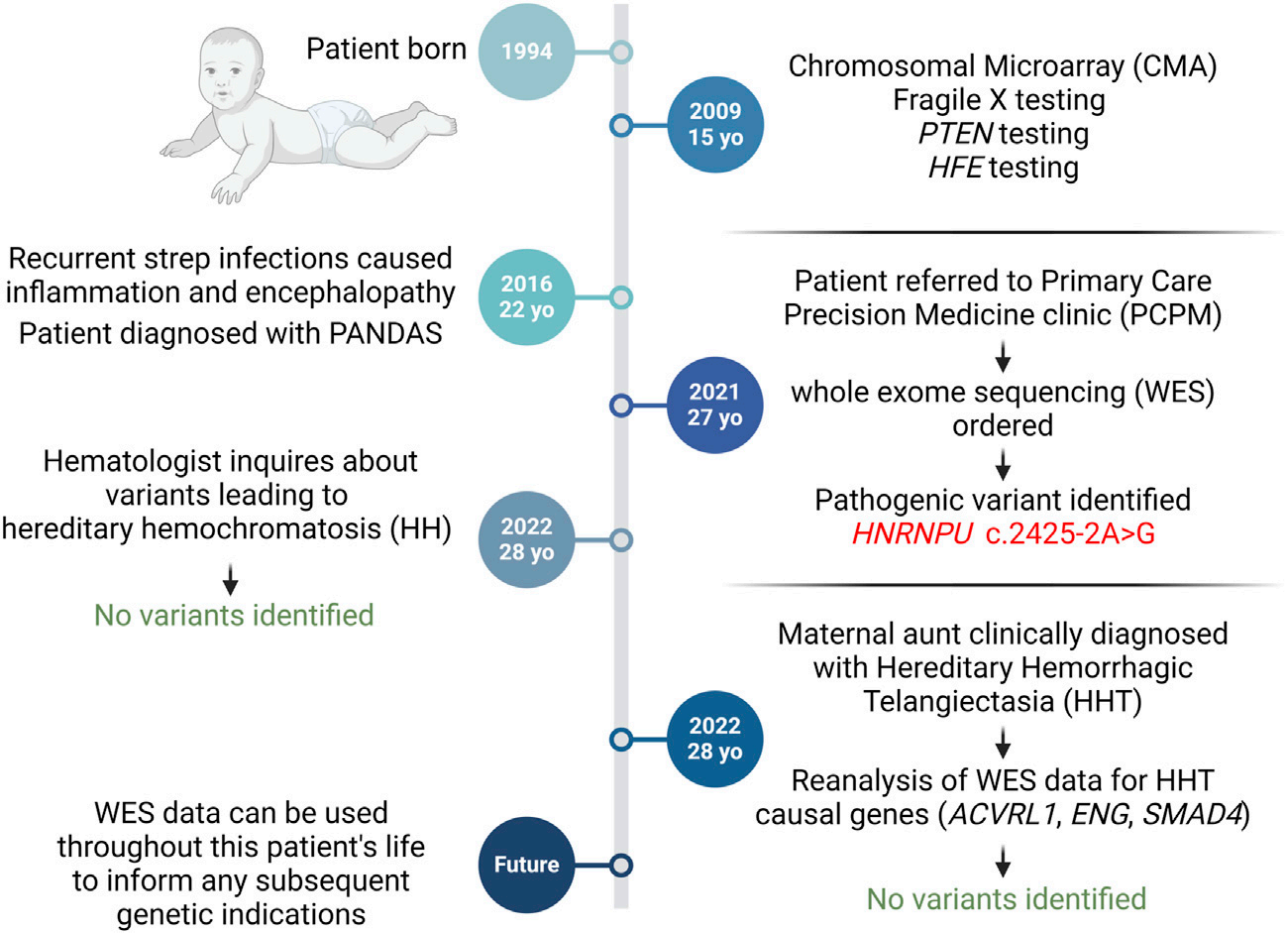
Diagnostic Timeline. At 15, the patient underwent genetic tests for neurodevelopmental symptoms without diagnostic yield. Referral to the PCPM clinic led to Whole Exome Sequencing (WES), unveiling a pathogenic HNRNPU mutation. WES data informed two subsequent genetic inquiries. Created with BioRender.com.

**FIGURE 3:**
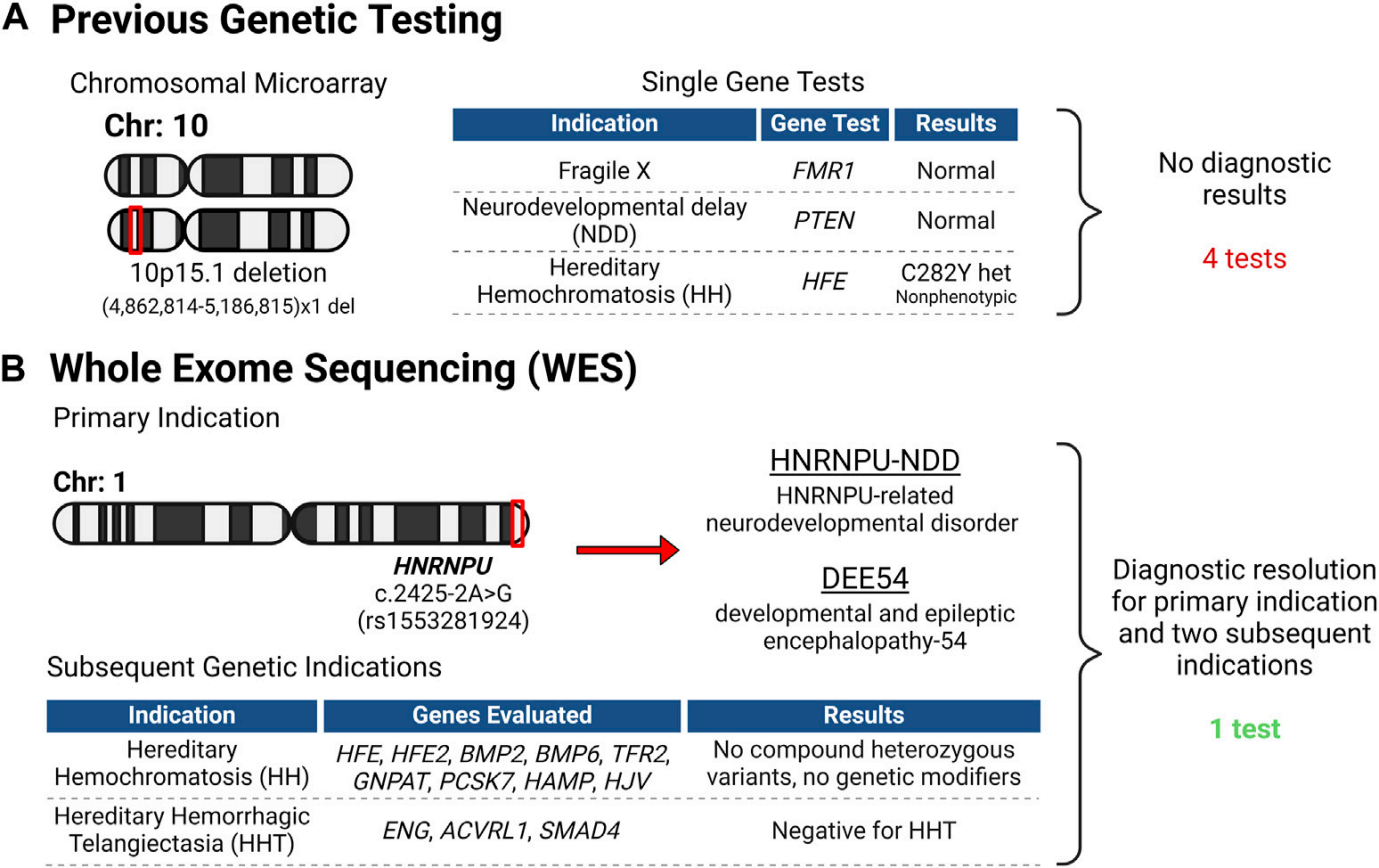
Genetic Testing Overview. **(A)** Before the patient’s evaluation at the PCPM clinic, numerous genetic tests were conducted, all proving uninformative. **(B)** Following intake at the clinic, Whole Exome Sequencing (WES) was initiated, leading to a conclusive diagnosis for the patient and her family. Importantly, the WES data was leveraged to address two more genetic inquiries, obviating the necessity for supplementary testing. Created with BioRender.com.

Previous genetic testing had been performed around age 15 (Figure 2), including fragile X testing and evaluation of *PTEN*, neither of which yielded actionable results. In addition, a microarray was performed, which revealed a 10p15.1 (4,862,814-5,186,815) ×1 deletion. However, this deletion was interpreted as not causal for the patient’s presentation. Based on the iron overload, the *HFE* gene was evaluated for common variants associated with hereditary hemochromatosis (HH). The patient was found to have a heterozygous mutation in the *HFE* gene (p.Cys282Tyr) that is often associated with HH; however this causality is linked to homozygous presence of the p. Cys282Tyr allele. Individuals with heterozygous p. Cys282TyrY *HFE* variant do not usually develop iron overload (Aguilar-Martinez et al., 2011). No other genetic findings were apparent with their previous genetic tests. Despite the multiple single gene and microarray tests, no explanation for the NDD, seizure history, and encephalopathy was identified (Figure 3A).

Upon consultation with the precision medicine clinic, WES was performed, which revealed a missense mutation in the *HNRNPU* gene (c.2425-2A>G; rs1553281924), which was reported as pathogenic and is associated with HNRNPU-related neurodevelopmental disorder (HNRNPU-NDD) and developmental and epileptic encephalopathy-54 (DEE54). This variant is absent from Gnomad but has been reported in a patient with HNRNPU-NDD (Depienne et al., 2017). OMIM reports of mutations in the *HNUNPU* gene with DEE54 (OMIM #: 617391) exhibit phenotypes that are consistent with the patient’s ID/ASD, seizure history, and encephalopathy (Figures 2, 3B), and this variant was considered diagnostic by the referring neurologist. This *HNRNPU* gene mutation is predicted to disrupt a canonical splice acceptor site and interfere with splicing, though the consequence on protein structure is unknown. Given its occurrence in the splice site of the last exon, it remains uncertain what spliced product would be produced and whether the resulting protein would be in-frame or out-of-frame, hence making it challenging to predict its potential for nonsense-mediated decay.

Another *HNUNPU* mutation has been reported that lies in a nearby genetic region to the variant described in this case report (Depienne et al., 2017). This variant (*HNRNPU* c.2425-3C>A) has been linked to DEE54 and shares similarities with the patient in our study, such as seizure age of onset and seizure triggers, albeit with much more severe symptoms. Our patient exhibits mild dysmorphic features, such as widely spaced eyes and high arched eyebrows. Her first febrile seizure occurred at 10 months old, with subsequent seizures often triggered by fever spikes. Developmentally, this individual displayed only minor delays; sitting commenced at 9 months, walking at around 17 months, with no notable delays in speech or behavior. However, following the PANDAS diagnosis in 2015, behavioral difficulties escalated, characterized by increased agitation, aggression, and expressive language challenges during episodes. Symptoms of OCD and ASD also intensified post-PANDAS diagnosis. These observed phenotypes align with previous reports of *HNRNPU* gene mutations and DEE54. Most patients with *HNRNPU* mutations experience seizures, with 91% occurring before the child turns 2 years old. Furthermore, half of the patients evaluated in this study had ASD and/or significant autistic features (Taylor et al., 2022). Obsessive-compulsive features have also been documented in several other reports (Bramswig et al., 2017; Depienne et al., 2017; Leduc et al., 2017; Durkin et al., 2020).

Although there is one other reported instance of this pathogenic mutation in ClinVar, the physical characteristics of individuals carrying this mutation have not been previously documented or reported. This novel variant represents a significant advancement in our medical understanding, contributing new insights to the field. In addition, three variants of uncertain significance (VUSs) were found in two genes (*MFN2* and *HSD17B4*), classified as possibly relating to the observed phenotype. While there was no change in treatment plan based on the genetic testing, these results finally provided answers to the patient and their family as to the underlying cause of their symptoms.

## Subsequent genetic inquiries

Over a year after WES was completed and based on the patient’s borderline hemochromatosis presentation, a physician from the Benign Hematology and Transfusion Medicine department (associated with the Vascular Medicine Institute) contacted the precision medicine clinic to reanalyze the WES data for either: 1) compound heterozygotes for other *HFE* mutations along with p. Cys282Tyr, such as point mutations that created a premature stop codon (p.Tyr52Ter; p. Glu168Ter; p. Leu270Ter; p. Ala271Ter; p. His341Ter); 2) other mutations affecting the protein sequence (p.Asp141Tyr; Lys166Asn); or 3) the presence of genetic modifiers of the HFE genes such as: *BMP2*, *BMP6*, *GNPAT*, *PCSK7* or variants in the genes for hepcidin (*HAMP*) or hemojuvelin (*HJV*). Reanalysis was completed on the data and no additional variants were found (Figures 2, 3B), thus the patient’s intermittent elevated serum iron concentration remains unexplained. Because the WES data were easily accessible to the precision medicine team, these results could be gathered within 1–2 days and required no additional samples or testing, decreasing diagnostic costs and increasing the utility of the initial WES testing.

Subsequently, the patient’s maternal aunt was clinically diagnosed with hereditary hemorrhagic telangiectasia (HHT), without genetic testing. Familial diagnosis of HHT is important as it presents an opportunity for cascade screening of close family members. The patient’s endocrinologist contacted the precision medicine clinic for WES reanalysis in light of this new family diagnosis. The main causal HHT genes were evaluated (*ACVRL1*, *ENG*, and *SMAD4*), which revealed no pathogenic variations (Figures 2, 3B). Though there are a few reported instances where large multi-exon deletions have been identified in HHT (Prigoda et al., 2006), the WES that was performed can detect deletions at least 1 kb in size. However, there remains a residual risk of missed small deletions and complex variants when the specific family genetic variant is unidentified. Nevertheless, the readily available ES results allowed for a swift reevaluation, minimizing, though not entirely ruling out, the need for individual assessment of the HHT genes. This efficient process underscores the utility of WES data, especially when organized systems allow for quick reanalysis as needed.

## Discussion

The utilization of WES in this case study provided a comprehensive and efficient means of diagnosing a complex neurodevelopmental disorder (NDD). By uncovering a pathogenic missense mutation in the *HNRNPU* gene, associated with HNRNPU-related neurodevelopmental disorder (HNRNPU-NDD) and developmental and epileptic encephalopathy-54 (DEE54), WES enabled a more precise understanding of the patient’s condition and provided answers for the patient and their family.

When promoting an alternative approach to patient care, cost-saving and time-saving justifications are needed to demonstrate how this paradigm is better than current practice. In this case, the clinical team was able to reanalyze the existing WES data to address subsequent indications, avoiding the need for additional tests. This streamlined approach not only expedites the diagnostic process but also contributes to cost reduction, which demonstrates the ongoing clinical utility of WES throughout the patient’s lifetime care (Klau et al., 2022).

Although this case represents the utility of WES, there are certain challenges that may arise in large-scale genetic testing. Identification of VUSs introduces a degree of uncertainty into the interpretation of WES results (Burke et al., 2022). While the variants identified in this case did not prompt a change in the treatment plan, they emphasize the ongoing challenge of interpreting genetic variations and the need for further research to elucidate the clinical significance of VUSs. Additionally, it's important to acknowledge that WES has its limitations, such as the inability to detect nucleotide repeat expansions, constraints in the size of copy number variations (CNVs) that can be detected, limited or poor coverage of some genes and/or exons, and difficulty in detecting variants in complex genomic regions. Looking ahead, these challenges may be addressed and overcome with advancements in WGS *in lieu* of WES as costs decrease, providing additional insights with detection of nucleotide repeat expansions, more uniform coverage across the genome, and the ability to detect CNVs of varying sizes.

Additionally, the success of repeat WES reanalysis relies heavily on effective interdisciplinary collaboration between various medical specialties, recognizing the importance of easily accessible prior genetic testing data that is well-documented in the patient’s chart. This not only streamlines the diagnostic process but also avoids unnecessary repeat testing, serving the patient more comprehensively across the lifespan (Rawlinson et al., 2021; Endrakanti and Gupta, 2023). This collaboration does not end at diagnostic resolution and must be carried forward to allow WES to address any subsequent genetic inquiries that arise. It is also crucial that patients (or their caretakers) understand the genetic test that has been performed and that they are well-informed on how this test can impact their care moving forward (Syurina et al., 2011; Gereis et al., 2022). The mother of our patient proved to be a huge advocate for her daughter and readily informed the other providers about the WES data, who were then able to contact the precision medicine clinic for WES reanalysis.

The case aligns with current medical literature advocating for the early use of WES in patients with neurodevelopmental disorders and demonstrates the utility of early use of WES in a patient’s diagnostic odyssey. A meta-analysis supporting the superior diagnostic capabilities of WES over traditional methods reinforces the growing consensus on the effectiveness of genomic sequencing in such cases (Vissers et al., 2017). The discussion touches upon the evolving landscape of genomic sequencing technologies, with a focus on the decreasing costs and increasing clinical utility of WES. This highlights a crucial take-away lesson: as sequencing technologies advance, the accessibility and affordability of WES may render it a primary diagnostic tool, minimizing the need for a battery of tests.

In conclusion, this case report demonstrates the strengths and current limitations of employing WES in the diagnostic journey of a patient with a complex neurodevelopmental disorder. Notably, if WES had been implemented earlier, this would have reduced the diagnostic odyssey and would have provided the family with answers over a decade sooner. Despite this diagnosis not altering the treatment, the patient and her family found comfort in having a final diagnosis and defined expectant management helping them better plan for the future. In addition, the patient is now eligible for gene-specific clinical trials. The ability to obtain a comprehensive diagnosis and the ongoing clinical utility of accessible WES data for reanalysis underscore the benefits of this approach. However, challenges such as the interpretation of variants of unknown significance and the dependency on interdisciplinary collaboration and patient health literacy should be acknowledged. The case contributes valuable insights to the evolving field of precision medicine and advocates for the early integration of WES to optimize patient care and reduce the burden on healthcare systems and payers.

## Patient perspective

Navigating the journey to a diagnosis for [Patient]’s complex symptoms was an arduous and exhaustive process for our family. Over the span of 5 years, we consulted numerous specialists across various medical disciplines, hoping to unravel the mystery behind [Patient]’s health challenges. Despite our efforts, the fragmented nature of medical care left us with more questions than answers, as each specialty offered only partial insights into [Patient]’s condition. Our feelings of frustration and uncertainty were alleviated when we received a referral to the Primary Care Precision Medicine (PCPM) clinic. Initially unfamiliar with precision medicine, we were hopeful as we learned about the approach and expertise of Dr. Massart. The prospect of a more holistic understanding of [Patient]’s health issues was both reassuring and promising. The initial consultation with Dr. Massart filled us with hope. Her compassionate and comprehensive approach, coupled with the promise of whole exome sequencing (WES), instilled confidence in us that we were on the right path towards finding answers for [Patient]. The anticipation during the wait for the results was mixed with nervousness and excitement, knowing that this could potentially lead us to the long-awaited diagnosis.

When we finally learned of [Patient]’s diagnostic variant, a profound sense of relief washed over us. Dr. Massart’s inclusive approach, involving [Patient] directly in the process, made the journey towards diagnosis feel less daunting and more empowering. [Patient]’s own words echoed our collective sentiment–the relief of finally having an answer outweighed any apprehension. Following the diagnosis, we proactively shared the WES results with other specialists, highlighting the importance of collaborative care and leveraging existing data to address subsequent inquiries. This collaborative effort underscored the critical role of informed patient advocacy in navigating the complexities of modern healthcare. Two moments stand out vividly amidst this journey: Dr. Massart’s ability to engage [Patient] directly, fostering a sense of ownership and empowerment in her healthcare journey, and her realistic yet hopeful approach, emphasizing the pursuit of knowledge and guidance regardless of the outcome. Looking ahead, our experience has fueled a desire for equitable access to WES for all individuals facing diagnostic challenges. The potential of WES to provide comprehensive insights and guide effective treatments is immense, and we believe that universal access to this technology can revolutionize healthcare outcomes for countless individuals and families. In conclusion, our journey exemplifies the transformative impact of precision medicine and underscores the importance of collaborative, patient-centered care in navigating complex medical conditions. We remain hopeful that our experience will contribute to broader discussions surrounding equitable access to genomic sequencing technologies, paving the way for a more inclusive and effective healthcare system for all.

## Scope statement

This case report outlines the diagnostic trajectory resolution of a 27-year-old female with a complex neurodevelopmental disorder (NDD) through Whole Exome Sequencing (WES). The manuscript underscores the role of WES in facilitating timely and cost-effective diagnoses, offering insights into the patient’s genetic landscape. The focus extends beyond the singular diagnostic event, emphasizing the broader implications for ongoing patient care, coordination, and the integration of WES data within the primary care setting. The manuscript aligns with the scope of the journal, emphasizing the clinical utility of WES, addressing challenges related to variant interpretation, and advocating for interdisciplinary collaboration. The relevance lies in the narrative’s contribution to the evolving field of precision medicine, showcasing the benefits of early WES integration, reduced diagnostic costs, and enhanced patient care coordination. The manuscript’s findings resonate with the journal’s emphasis on advancing genomic sequencing technologies and their clinical applicability, thereby making it a suitable fit for consideration in the journal’s platform.

## Data Availability

The original contributions presented in the study are included in the article/Supplementary material, further inquiries can be directed to the corresponding author.
